# Effect of human immunodeficiency virus self-testing (HIVST) on HIV and STI testing uptake among men who have sex with men (MSM): A systematic review and meta-analysis of randomized controlled trials

**DOI:** 10.12669/pjms.41.6.11774

**Published:** 2025-06

**Authors:** Safdar Kamal Pasha, Usman Ali, Ramesh Kumar

**Affiliations:** 1 Safdar Kamal Pasha National Professional Officer for HIV, Hepatitis and STI, World Health Organization, PhD Scholar (Public Health), Health Services Academy, Pakistan; 2 Usman Ali Public Health Specialist, Postgraduate Resident, King Edward Medical University, Lahore, Pakistan; 3 Ramesh Kumar Professor of Public Health, Health Services Academy, Islamabad, Pakistan

**Keywords:** Men who have sex with men, HIV self-testing, Traditional diagnostic tests, Sexually transmitted infections

## Abstract

**Objectives:**

The objective of this review was to assess the effectiveness of HIVST in terms of increasing HIV testing, early detection of HIV, and sexually transmitted infections among MSM via HIVST.

**Methods:**

We conducted a systematic review and meta-analysis of randomized controlled trials. We searched PubMed/MEDLINE, EMBASE, Cochrane Central, and clinicaltrials.gov. Data were extracted from May 2013- May 2024. RCTs comparing HIVST with traditional HIV testing among MSM were included in the review. Data was analyzed using Review Manager version five.

**Results:**

A total of fifteen randomized controlled trials (RCTs) were identified and included in the qualitative synthesis, and thirteen were included in the meta-analysis. The mean difference in HIV test results between the HIVST group and the traditional HIV testing group was 2.07 (95% confidence interval (CI) 1.54--2.60). The odds ratio for the detection of incident HIV in the HIVST group was 1.55 (95% confidence interval 1.02–2.37). The odds ratio of HIV test uptake from eleven RCTs was 5.17 (95% confidence interval 1.90—14.06) for HIVST compared with traditional HIV testing. Pooled analysis revealed that the odd ratio of STI test uptake was 0.86 among the HIVST group compared with the traditional HIV testing group (P <.02).

**Conclusions:**

HIVST is associated with increased uptake of HIV testing and an increased mean number of HIV tests among MSM. It also leads to increased detection of HIV. However, STI testing decreases with HIVST, likely because of facility-based HIV testing in the control arm.

## INTRODUCTION

Human immunodeficiency virus self-testing (HIVST) is a rapid diagnostic test in which people themselves can perform sampling and interpretation with little or no assistance from trained healthcare providers.^1^ Based on evidence for test performance and its potential impact in terms of overcoming healthcare access barriers, the World Health Organization (WHO) recommends HIVST as a testing strategy to increase the number of people who are aware of their HIV status.^2^ HIVST can be a game changer and an empowering tool for people at risk for HIV. HIVST decreases barriers to accessing HIV testing, which may stigmatize key populations and people at risk.^3^ Thus, HIVST can be used to increase the coverage of HIV testing. Intervention is cost-effective and can lead to earlier detection of HIV and a need for treatment.^3^

In a network meta-analysis conducted in 2021, it was revealed that there is an increased uptake of HIV self-tests compared with rapid diagnostic tests for HIV conducted by healthcare workers (hereby called traditional HIV testing).^1^ Various HIVST distribution strategies are used globally. These include the provision of HIVST kits in the drop-in center or by outreach workers of an HIV prevention site and provision via online orders. In fact, HIVST was provided via the internet and courier-based dispensation in various countries during the COVID-19 lockdown 4-7

Globally, 0.7% of the adult population is living with HIV; however, the prevalence is higher among key populations. The global prevalence of HIV among men who have sex with men (MSM) is 7.5%.^8^ MSM also face significant challenges related to accessing confidential HIV services and face stigma at healthcare service delivery points, which prevents them from accessing HIV testing.^9^ In fact, HIVST has been proven to be a cost-effective intervention for key populations in low-HIV-incidence settings.^3^ The current systematic review and meta-analysis aims to gauge the effectiveness of HIVST in terms of increasing HIV testing, early detection of HIV, and uptake of sexually transmitted infection (STI) testing among MSM using HIVST compared with traditional rapid diagnostic HIV testing (RDT) administered by healthcare workers.

## METHODS

The methodology of this systematic review follows the Methodological Expectations of Cochrane Intervention Reviews (MECIRs).^10^ The review was registered at the International Prospective Register of Systematic Reviews (PROSPERO) (CRD42024527359).

### Search strategy:

We searched PubMed/MEDLINE, EMBASE, Cochrane Central, and clinicaltrials.gov via advanced search options. The search terms included the following MeSH items:

*‘men who have sex with men OR MSM OR gay man OR gay men OR same sex behavior OR bisexual man OR bisexual men **AND** HIV OR AIDS OR sexually transmitted disease OR sexually transmitted infection OR syphilis OR chlamydia OR gonococcal’*

Data were extracted from Jan 2013--May 2024. Book chapters, conferences, proceedings, reviews, and original articles were identified. Manual searches of review articles identified through the above search strategy were also performed. We also requested that colleagues at the World Health Organization Offices, the United Nations Joint Program on HIV/ AIDS and community stakeholders share any relevant gray literature related to review questions. The bibliographic data were imported into Rayyan (www.rayyan.ai). In the first stage, duplicates were removed by reviewer one, followed by screening of publication titles by reviewer one. The abstracts of the screened articles were reviewed by reviewer one and reviewer two against the PICO questions mentioned below. Any disagreements were referred to Reviewer three for a final decision on the inclusion or exclusion of the article. The full texts were reviewed by reviewers one and two, followed by an assessment of the risk of bias via risk of bias-2 (RoB) for randomized trials by reviewers one and two, and disagreements were referred to by reviewer three.

### Inclusion criteria:

- Two authors independently included research articles reporting a randomized clinical trial AND meeting the PICO criteria listed below:

### PICO Questions:

- **Population:** Men who have sex with men
- **Intervention:** HIV self-testing
- **Comparator:** traditional rapid diagnostic test (RDT) conducted by a trained healthcare worker
- **Outcomes:**
  - Frequency of HIV tests using HIVST compared with conventional HIV tests at study’s last follow-up period and at twelve months
  - Detection of HIV at months after induction in the two arms at study’s last follow-up and at twelve months
  - Uptake of HIVST compared with traditional HIV testing during at study’s last follow-up and at twelve months
  - Uptake of test other sexually transmitted infections at study’s last follow-up and twelve months

### Data analysis:

We conducted data analysis via the Cochrane Review Manager Version 5.4. Data analysis was conducted and reported according to the Preferred Reporting Items for Systematic Review and Meta-analysis (PRISMA) guidelines. The mean differences were calculated for both the primary and secondary outcomes via a random effects model with a 95% confidence interval. Forest plots were generated and reported. Confidence in findings was evaluated via Grades of Recommendation, Assessment, Development, and Evaluation (GRADE).^11^

### Findings:

Using the search strategy, a total of six hundred and seventy studies were identified through databases, and fourteen articles were identified through manual searching of the bibliography of published articles. After the removal of duplicate studies, six hundred and fifty studies were included for initial screening, followed by a review of the full texts of twenty-five articles for assessing eligibility against PICO questions. Fifteen randomized controlled trials (RCTs) were identified and included in the qualitative synthesis, and thirteen were included in the meta-analysis. The characteristics of these RCTs are given in Table-I. None of the studies reported the uptake of STI tests at one year, whereas only two reported the uptake of STI tests at different time points.

**Table-I:** Characteristics of the included studies.

Sr. No.	Authors and year of publication	Country/Region	Sample size	HIVST distribution strategy	Outcome
*Outcomes reported at less than 3 months since randomization*					
1.	Wirtz 2022^19^	Myanmar	577	Delivered via a community HIV testing center	8.9% in the HIV Testing Service (HTS) were newly diagnosed (self-reported) with HIV compared with 14.6% in HIVST group. 54% in HTS vs. 62% in HIVST completed HIV test.
*Outcomes reported at 3 months since randomization*					
2.	Merchant 2018^20^	United States of America	425 (three groups)	Internet-based distribution of HIVST and mail-in blood sample	Three groups were made with the primary outcome of HIV test completion. Oral HIVST kits were provided to one group, whereas Home-based mail-in blood sample collection conventional HIV test was provided to the second group, while standard facility-based HIV testing was provided to the control group. HIV test completion was highest in the oral HIVST group, 66.2%, followed by the facility-based HIV testing group, 56.0%, and lowest in the home-based mail-in blood sample collection conventional HIV test 40.1%
3.	Zhu 2019^21^	China	100 (two groups)	By a healthcare worker	The intervention group was given access to mobile-based application, control group had access to HIVST but not the mobile application. Intervention Intervention group had higher rates of HIV testing overall RR=1.99, and via oral HIVST RR=2.17.
4.	Young 2022^22^	United States of America	900 (two groups)	Distribution after online outreach by peers	Participants in the intervention group were 1.47 times likely to have an HIVST in last three months. There was no significant difference in sexual risk behaviors among two groups.
5.	Rodger2022^18^	United Kingdom	10111 (two groups)	Internet-based distribution	The primary outcome was HIV diagnosis after three months of enrollment. 19/6041 in the HIVST group had an HIV diagnosis at three months as compared to 15/4062 in the control group. The results were not statistically significant. Frequency of HIV testing at three months was the secondary outcome. 97% of HIVST had an HIV test in three months compared with 43% in control group.
*Outcomes reported at 6 months since randomization*					
6.	Wang 2018^23^	Hong Kong	430 (two groups)	In-person appointments during which a three-minute video was shown, followed by mailing of HIVST. Supervised test was performed either online or in real time.	Participants in the HIVST group had more HIV tests at 6 months, RR 1.77 (95% confidence interval 1.54 to 2.03) compared to the control group.
7.	Cheng 2021^24^	China	491	Blood-based HIVST with access to a video tutorial provided to participants	77.4% of MSM in HIVST, 69.5% of MSM in facility-based HIV testing had taken an HIV test during the follow-up period.
8.	Wray 2018 25	United States of America	65 (three groups)	Distribution of HIVST with or without eTEST application. In the control group, reminders but not HIVST was provided	The HIVST (with or without eTEST application) group was 4.4 times more likely to have an HIV test as compared to controls who were only sent HIV test reminders. The HIVST groups (with or without eTEST application) have a significantly greater number of tests at 3- and 6-month follow-up.
9.	Zhou 2023^26^	China	620 participants (two groups)	Internet-based distribution	The intervention group had a higher uptake of HIV tests at 3 months and 6 months, with adjusted risk difference of 36.7 and 26.7, respectively
*Outcomes reported at twelve months since randomization*					
10.	Jamil 2017^27^	Australia	362 (two groups)	Distribution via sexual health clinics	The primary outcome was the frequency of HIV tests among both groups. MSM in the HIVST group had 4 tests per year compared to 1 in non non-HIVST group. The mean new HIV infections in the HIVST group was 4/182 vs. 1.9/180 in the control group at twelve months.
11.	MacGowan 2020^28^	United States of America	2665 (two groups)	Internet based distribution	The primary outcome was the frequency of HIV testing and the number of new HIV infections diagnosed in both groups. The self-testing group reported 5.3 tests compared to 1.5 in the control group over one year. Twenty-five new HIV infections were diagnosed in the self-testing group compared to eleven in the control group.
12.	Zhang 2020^29^	China	230 MSM (two groups)	In-person distribution with electronic guidance on how to use HIVST	The primary outcome was the number of HIV tests, which was higher at 2.65 tests (mean per person) in the HIVST group vs. 1.31 in the control group.
13.	Frye 2021^30^	United States of America	200 participants (two groups)	In-person distribution	Behavioral intervention delivered by peers to support HIV screening. Significantly increased HIVST uptake at 3 and 6 months between intervention and control groups with odds ratios of 2.29 and 1.94, respectively. However, no significant increase at 9 and 12 months between the two arms.
*Outcomes reported after twelve months since randomization*					
14.	Katz 2018^17^	United States of America	230 (two groups)		The primary outcome was the number of HIV tests per person. The mean HIV test per person in the HIVST group was 5.3 at fifteen months compared with 3.6 in the control group. In terms of new HIV diagnosis, however, men in HIVST had fewer STI tests, 2.3 vs. 3.2 in the control group. Four MSM in the HIVST group were newly diagnosed with HIV infection compared with two in the control group. Group differences were not significant.
15.	Zhang 2021^31^	Australia	343 for year 2 (two groups	Distribution via sexual health clinics	Follow-up data of FORTH trial for year two. The mean number of tests per person in the HIVST arm was 3.7 for year 2 and 3 for year 1

**Fig.1:**
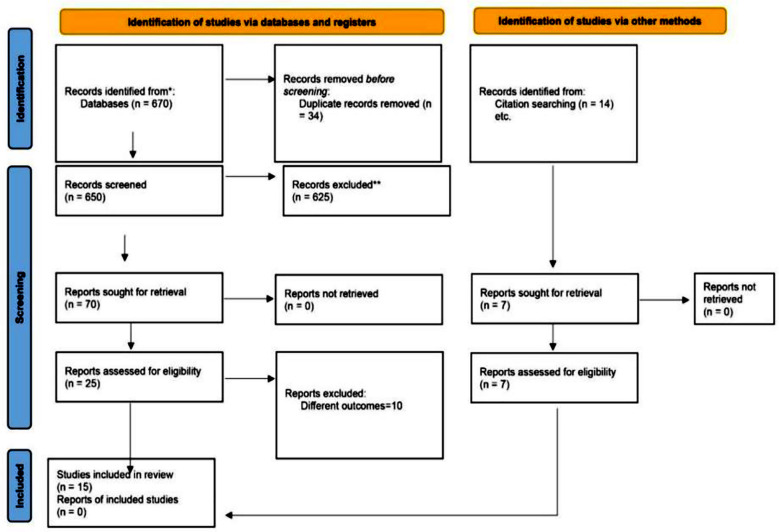
PRISMA flow diagram of literature search.

The study flow diagram is shown in Fig.1. The risk of bias was determined via Cochrane’s risk of bias (RoB) tool-2 by two independent authors, and discrepancies were settled by referring to a third author. The tool proposes an algorithm for judging bias across five domains.^12^ RoB assessments are given as supplements to this article. A forest plot for the mean number of HIV tests among the HIVST and traditional HIV testing groups at study’s last follow-up is shown below. On the basis of pooled results from six different randomized controlled trials, the mean difference in HIV test results between the HIVST group and the traditional HIV testing group was 2.07 (95% confidence interval (CI) 1.54--2.60), which was statistically significant. Fig.3 shows the means of the HIV test results among the HIVST and traditional HIV testing groups at twelve months. Only three studies reported mean HIV test results at 12 months. The pooled results revealed a mean difference of 1.92 (95% confidence interval 1.60-2.24s), which was statistically significant. The forest plot in Fig.4 shows the detection of incident HIV infection among the HIVST and traditional HIV testing arms at study’s last follow-up. The results were pooled from six RCTs. The odds ratio of detection of incident HIV in the HIVST group were 1.55 (95% confidence interval 1.03–2.37).

**Fig.2:**
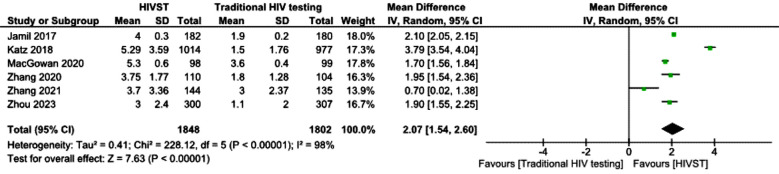
Mean number of HIV tests among the HIVST and traditional HIV testing groups.

**Fig.3:**
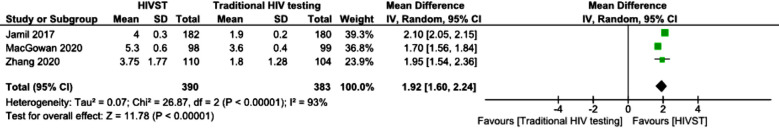
Mean number of HIV tests among the HIVST and traditional HIV testing groups at twelve months.

**Fig.4:**
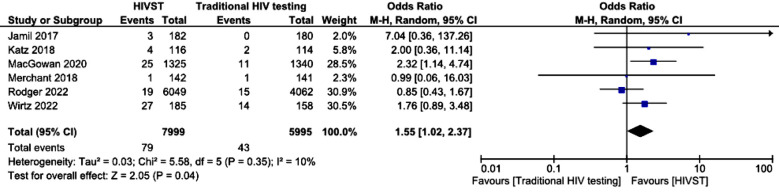
Detection of incident HIV infections among the HIVST and traditional HIV testing groups.

We performed a subgroup analysis of the detection of incident HIV infections in only studies that reported 12 months outcomes. Only two studies reported outcomes at twelve months. The results favored HIVST, with an odds ratio of 2.47 (95% CI 1.23--4.74), which was statistically significant (p <.01). The results of a total of eleven RCTs were extracted to determine the odds ratio (OR) of the use of HIV tests among the HIVST and traditional HIV testing groups at study’s last follow-up. The odds ratio from pooled analysis was 5.17 (95% confidence interval 1.90—14.06; p <.00001). Subgroup analysis of HIV test results at twelve months revealed that the uptake was significantly different between the HIVST group and the traditional HIV testing group at twelve months, with an odds ratio of 6.17 (95% confidence interval 1.36–28.10) (Fig.7).

**Fig.5:**
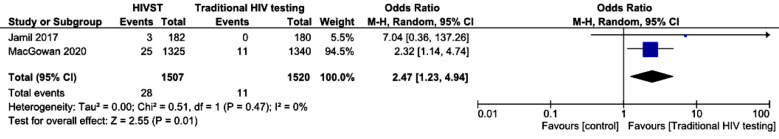
Detection of incident HIV infections among the HIVST and traditional HIV testing groups at twelve months.

**Fig.6:**
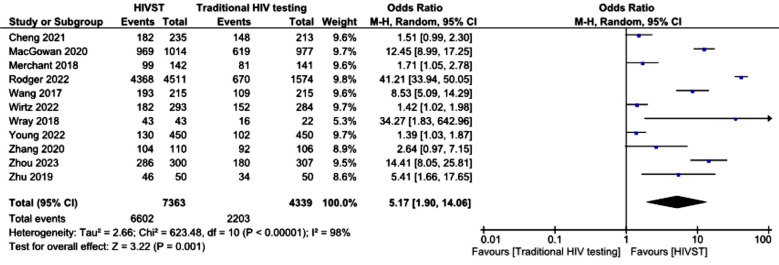
Uptake of HIV testing among the HIVST and traditional HIV testing groups.

**Fig.7:**
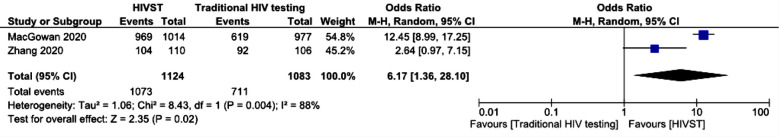
Uptake of HIV testing among the HIVST and traditional HIV testing groups at twelve months.

**Fig.8:**
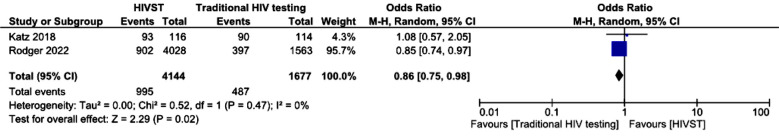
Uptake of STI tests among the HIVST and traditional HIV testing groups

Only two RCTs mentioned STI test uptake in HIVST RCTs, and none reported outcomes at twelve months. Pooled analysis revealed that the odds ratio of STI test uptake was 0.86 among the HIVST group compared with the traditional HIV testing group (p <.02). We also conducted an evaluation of the findings via the GRADE methodology. A summary of the findings is given in Table-II. On the basis of evidence, there is high certainty that HIVST increases HIV testing uptake and the mean number of tests. However, there is very little certainty as to whether it increases the uptake of HIVST at twelve months and its effect on STI testing uptake.

**Table-II:** Summary of findings obtained via the GRADE methodology.

Outcomes	Anticipated absolute effects* (95% CI)		Relative effect (95% CI)	№ of participants (studies)	Certainty of the evidence (GRADE)	Comments
	Risk with traditional HIV testing	Risk with HIV self-testing				
Mean no. of HIV test		MD 2.07 higher (1.54 higher to 2.6 higher)	-	3650 (6 RCTs)	⨁⨁⨁⨁ High	HIV self-testing results in large increase in mean no. of HIV test.
Mean no. of HIV tests at twelve month (Mean HIV tests at twelve months)	-	SMD 2.62 SD higher (1.45 higher to 3.79 higher)	-	2567 (3 RCTs)	⨁⨁⨁⨁ High	HIV self-testing results in large increase in mean no. of HIV tests at twelve months.
Detection of incident HIV infections	7 per 1,000	11 per 1,000 (8 to 16)	OR 1.58 (1.09 to 2.30)	13994 (6 RCTs)	⨁◯◯◯ Very Low^a,b^	HIV self-testing may increase detection of incident HIV infections slightly.
Detection of incident HIV infections at twelve months	7 per 1,000	18 per 1,000 (9 to 35)	OR 2.47 (1.23 to 4.94)	3027 (2 RCTs)	⨁⨁◯◯ Low^c^	HIV self-testing may increase/have little to no effect on detection of incident HIV infections at twelve months but the evidence is very uncertain.
Uptake of HIV testing	508 per 1,000	842 per 1,000 (662 to 935)	OR 5.17 (1.90 to 14.06)	11702 (11 RCTs)	⨁⨁⨁⨁ High^d^	HIV self-testing results in large increase in uptake of HIV testing.
Uptake of HIV test by twelve months	657 per 1,000	922 per 1,000 (722 to 982)	OR 6.17 (1.36 to 28.10)	2207 (2 RCTs)	⨁◯◯◯ Very Low^c,d^	HIV self-testing may increase/have little to no effect on uptake of HIV test by twelve months but the evidence is very uncertain.
Uptake of STI test	290 per 1,000	260 per 1,000 (235 to 286)	OR 0.86 (0.75 to 0.98)	5821 (2 RCTs)	⨁◯◯◯ Very Low^c,e^	HIV self-testing may reduce/have little to no effect on uptake of STI test but the evidence is very uncertain.

* The risk in the intervention group (and its 95% confidence interval) is based on the assumed risk in the comparison group and the relative effect of the intervention (and its 95% CI).

CI: confidence interval; MD: mean difference; OR: odds ratio; SMD: standardized mean difference

GRADE Working Group grades of evidence

High certainty: we are very confident that the true effect lies close to that of the estimate of the effect.

Moderate certainty: we are moderately confident in the effect estimate: the true effect is likely to be close to the estimate of the effect, but there is a possibility that it is substantially different. Low certainty: our confidence in the effect estimate is limited: the true effect may be substantially different from the estimate of the effect.

Very low certainty: we have very little confidence in the effect estimate: the true effect is likely to be substantially different from the estimate of effect.

***Explanations***

a. Five RCTs were included; 2 had a high risk of bias, and 1 had some concerns. b. All RCTs included had nonsignificant detection of incident HIV infection except one RCT. c. One RCT reported nonsignificant differences between the HIVST and traditional HIV testing groups, and the other RCT reported significant differences. d. Three of the eleven included RCTs had a serious overall risk of bias. e. Two RCTs included in the analysis; one had a high risk of bias.

## DISCUSSION

In our review, we found that the mean difference in the frequency of HIV testing between the two groups was 2.4 at the end of the twelve-month period. Johnson et al. conducted a meta-analysis on the effect of HIVST on the use of HIV testing. The meta-analysis pooled the results of all populations. The analysis revealed that HIVST doubles HIV testing uptake among men.^13^ More recently, Witzel and colleagues conducted a meta-analysis exploring the impact of HIVST on testing frequency among all key populations. According to Witzel et al., HIVST increased HIV testing uptake by 1.45 among key populations.^14^

In terms of the detection of HIV, the likelihood of detecting incident HIV was 2.40 times greater than that of traditional RDT. However, the confidence in the findings is very low based on GRADE methodology. Witzel et al. previously reported no group differences for HIV detection pooled for key populations among HIVST takers vs. the comparator group.^14^ Similarly, an RCT from the UK reported no difference in the number of HIV-diagnosed patients between the two groups.^15^ Early diagnosis and prompt treatment are pillars of secondary prevention. Early diagnosis of HIV leads to early initiation of antiretroviral therapy (ARV).^16^ HIVST allows self-sampling and testing and thus can increase the use of HIV testing in this population, as seen in our meta-analysis. HIVST also leads to a significant increase in the distribution of HIVST kits among partners/peers (secondary distribution).^14^ Thus, it provides a window of opportunity for reaching hard-to-reach MSM populations who may not be in contact with HIV services. Witzel et al. reported increased uptake of HIVST by using online or mail distributions, whereas the uptake was not significantly different if HIVST was provided at a facility.^14^ This highlights that HIVST is effective in increasing the use of HIV testing among online-based communities, such as MSM, who have different barriers to accessing HIV care than do those who visit facilities.

We included outcomes on the use of tests for sexually transmitted infections. Only two RCTs reported outcomes for this indicator. We conclude that there was a lower odds of STI test uptake among the HIVST group. Katz et al. reported data from their RCT on STI testing and reported that men in HIVST had fewer STI tests (2.3 vs. 3.2 in the control group).^17^ In the same RCT, MSM in the HIVST group had a 5.4% prevalence of STIs in HIVST compared with 12.2% in the control group at the end of the study, i.e., nine months. The increase in STI testing in the control group in this RCT may be due to an increase in facility visits in the control group to obtain an HIV test, whereas HIVST can be mailed. The same RCT reported an odds ratio of 1.07 for condomless anal intercourse in the HIVST group, but the group difference was not statistically significant.^17^ To date, the largest trial on HIVST has been carried out in the UK, with the inclusion of over 10,000 MSM.^18^

The study revealed that 97% of the MSM in the HIVST group completed any HIV test, whereas 43% of the MSM in the HIVST group completed any HIV test at baseline. The STI test rate was 25% in the control group compared with 22% in the HIVST group, which was statistically significant. Subgroup analysis revealed that among those who completed an HIV test in both groups, 56% in the non-HIVST group and 23% in the HIVST group completed the STI test. Thus, we can conclude that although HIVST can increase the frequency of HIV testing, it may lead to decreased testing of STIs, likely because self-testing for STIs is not available except for syphilis, and STI testing is facility-based.

### Limitations

The limitation of this review is that only a few RCTs reported outcomes at twelve months. Among the fifteen different RCTs identified, six were conducted in the USA, four in China, and one each in the UK, Hong Kong, Myanmar, and Australia. There is no evidence from Latin American, South Asian, or African countries on HIVST among MSM. In addition, the results of the RCTs did not report results uniformly, thus leading to difficulty in comparing studies and a lack of inclusion in the meta-analysis. Furthermore, STI testing and detection were not uniformly reported at various time points.

## CONCLUSION

This review revealed that HIVST is associated with increased uptake of HIV testing and an increased mean number of HIV tests among MSM (GRADE assessment, high certainty evidence). It also leads to increased detection of HIV (GRADE assessment, low certainty evidence). However, STI testing decreases with HIVST, likely because of facility-based HIV testing in the control arm (GRADE assessment, very low certainty evidence). In addition, the evidence base is limited to a few countries. Further research on STI test uptake and program interventions for repeated testing reminders to HIVST users is necessary to fully leverage the potential of HIVST among MSM.
